# Rare and Complex: lessons from a cohort of patients with Atypical Hemolytic Uremic Syndrome

**DOI:** 10.1590/2175-8239-JBN-2021-0066

**Published:** 2021-05-28

**Authors:** Lilian Monteiro Pereira Palma

**Affiliations:** 1 Universidade Estadual de Campinas, Faculdade de Ciências Médicas, Departamento de Pediatria, Campinas, SP, Brasil.

In this issue of the Brazilian Journal of Nephrology, Maximiano et al*.* 1 in the paper intitled "Genetic atypical hemolytic uremic syndrome in children: a 20-year experience from a tertiary center" describe five pediatric patients with atypical Hemolytic Uremic Syndrome (aHUS) and positive genetic findings over a period of 20 years in Porto, Portugal. All of them had the first manifestation before 2 years of age and had complete remission with plasma exchange (n=4) or supportive measures (n=1). Two patients had frequent relapses, one of whom progressed to Chronic Kidney Disease (CKD) and hypertension.

The small number of patients is a common finding in studies of rare diseases and justifies the creation of regional, national, and even global registries. Although the paper focuses on pediatric cases, it is important to acknowledge that aHUS also debuts in the adult age^2^.

The first association between aHUS and the alternative complement defect was described in 1981^3^ in siblings from consanguineous parents with aHUS and decreased blood levels of Complement Factor H (CFH). Two decades later, the team lead by T. Goodship^4^ revealed the association between aHUS and chromosome 1q32, which contains the genes for complement regulators. In the following years, defects in other components of complement regulators of the alternative pathway have been found (inactivating mutations in genes that encode complement regulating proteins such as *CFH, CFI*, and *MCP* or gain-of-function mutations in genes that encode complement activating proteins such as *C3* and *CFB*). Factor H autoantibodies (FHAA) are also associated with aHUS, typically in children who are homozygous deleted for the *CFHR1* gene, a member of the *CFH* gene family. The frequency of this deletion allele varies across the globe from a high of over 50% in Nigeria to very rare in South America and Japan. How deletion of *CFHR1* leads to development of FHAA is complicated and may involve slight differences in structural conformation of Factor H related protein 1 and Factor H itself, as well as an individual's susceptibility to the development of autoantibodies in general^5^.

In the cohort described by Maximiano et al., two children presented with CFH variants. Other two patients had variants in the CFHR3/1 and CFHR3 genes, which encode proteins related to Factor H and are usually considered Variants of Unknown Significance (VUS). Since 2015, determination of pathogenicity of identified variants follows the American College of Medical Genetics and Genomics (ACMG) guidelines^6^. Accurate classification is paramount to clinical care and remains one of the main challenges today. As such, testing is best done in laboratories with specialized expertise in complement genetics. Since this information is not available in the mentioned paper, we may infer from the genetic variants described in table 2 that only two patients had pathogenic variants in CFH, although in heterozygosis. Data from The Global aHUS Registry^7^ with more than 800 patients enrolled confirmed that adults have worse kidney outcome than children, as well as patients carrying CFH pathogenic variants.

Non-complement genes such as *MMACHC* (methylmalonic aciduria cblC type with homocystinuria), *INF2* (inverted formin 2), and *DGKE* in homozygosis (diacylglycerol epsilon) have been associated with aHUS. One patient in this Portuguese cohort presented a homozygous mutation in DGKe in addition to a variant in CFHR3, whose pathogenicity was not described.

In light of incomplete genetic penetrance (estimated to be 50%), the current hypothesis is that the development of aHUS requires "two hits", which is a combination of genetic background and a trigger, most commonly an infection (as noticed in this paper), with potential management implications. One of the main challenges is to differentiate aHUS unmasked by a trigger from secondary causes of Thrombotic Microangiopathy (TMA) - those in which the underlying cause has a direct role in endothelial damage. In a recent review^8^, severity and extent of genetic complement abnormalities in secondary TMA was shown to be variable. Malignant hypertension and pregnancy-associated TMA are more likely to be associated with genetic complement abnormalities, and appear similar to those in aHUS, whereas autoimmune diseases and drug-associated TMA are less likely to have genetic complement abnormalities. Genetic complement defects in infection-associated TMA are variable, and infections may trigger aHUS. The early use of complement inhibition in patients with secondary TMA refractory to traditional therapy may be attempted provided there is significant organ dysfunction.

In the cohort described by Maximiano et al., complete remission was achieved in all patients: in four patients after plasma exchange and in one patient after supportive measures. Only one patient needed transient dialysis (with a C3 variant). Relapses happened in two patients: one patient with CFH and one patient with DGKe - this latter patient progressed with systemic hypertension and CKD despite chronic plasma therapy, revealing an inexorable outcome due to still unknown pathophysiologic mechanisms^9^. No patient received a complement inhibitor, and the fact that remission was attained with plasma exchange or supportive measures may be attributed to either the small cohort or lack of proven pathogenicity of the genetic variants. It is important to point out that guidelines suggest eculizumab as first line treatment^10^ in children with highly suspicious aHUS. Nevertheless, since this drug is not available worldwide, supportive measures and plasma therapy may be lifesaving, although less effective for kidney-saving.

It would be important to compare these outcomes with a broader population of patients with aHUS, and it has become clear that local and regional registries are key to shed light on the genetic background and outcomes of this disease. This paper brings a great contribution to still unsolved questions in aHUS: how can a precise diagnosis be made and how to best tailor the therapeutic approach.
